# OmicsARules: a R package for integration of multi-omics datasets via association rules mining

**DOI:** 10.1186/s12859-019-3171-0

**Published:** 2019-11-08

**Authors:** Danze Chen, Fan Zhang, Qianqian Zhao, Jianzhen Xu

**Affiliations:** 1 Computational Systems Biology Lab, Department of Bioinformatics, Shantou University Medical College (SUMC), No.22, Rd. Xinling, Shantou, China; 2 Guangdong Provincial Key Laboratory for Breast Cancer Diagnosis and Treatment, Cancer Hospital, Shantou University Medical College (SUMC), Shantou, 515041 China

**Keywords:** OmicsARules, Multi-omics experiments, Association rules, R package, Data integration

## Abstract

**Background:**

The improvements of high throughput technologies have produced large amounts of multi-omics experiments datasets. Initial analysis of these data has revealed many concurrent gene alterations within single dataset or/and among multiple omics datasets. Although powerful bioinformatics pipelines have been developed to store, manipulate and analyze these data, few explicitly find and assess the recurrent co-occurring aberrations across multiple regulation levels.

**Results:**

Here, we introduced a novel R-package (called OmicsARules) to identify the concerted changes among genes under association rules mining framework. OmicsARules embedded a new rule-interestingness measure, *Lamda3*, to evaluate the associated pattern and prioritize the most biologically meaningful gene associations.

As demonstrated with DNA methlylation and RNA-seq datasets from breast invasive carcinoma (BRCA), esophageal carcinoma (ESCA) and lung adenocarcinoma (LUAD), *Lamda3* achieved better biological significance over other rule-ranking measures. Furthermore, OmicsARules can illustrate the mechanistic connections between methlylation and transcription, based on combined omics dataset. OmicsARules is available as a free and open-source R package.

**Conclusions:**

OmicsARules searches for concurrent patterns among frequently altered genes, thus provides a new dimension for exploring single or multiple omics data across sequencing platforms.

## Background

Disease initiation and progression often result from multiple aberrations at multiple regulation dimensions. The improvements of high throughput technologies have enabled them to be precisely characterized at epigenetic, genomic, transcriptomic, proteomic and metabolomic levels [1–3]. While this opens the door to a systems-based research approach, there is an urgent demand of novel methods to better illustrate the underlying mechanistic connections within or across different omics datasets.

Previously, analyzing the cancer genomic data have identified associated mutations among a few genes. For example, George et al. have conducted whole genome sequencing on 110 small cell lung cancers (SCLC). They found TP53 and RB1 are universally mutated in all but two cases, which supported TP53 and RB1 follow the classical discrete ‘two-hit paradigm’ pattern of Knudson type tumor suppressors in SCLC [4]. Indeed, integrative analysis of multi-modal datasets of the same cancer tissue further revealed that some genes often concurrently altered at multiple regulation levels [5, 6]. For instance, adult cases of de novo acute myeloid leukemia were analyzed using genome sequencing along with RNA/microRNA sequencing and DNA-methylation chip. The multi-omics datasets showed that gene fusion events were correlated with specific patterns of mRNA expression, and the occurrences of specific mutations were associated with some expression signatures [6]. Importantly, the co-occurrence pattern among significantly mutated genes, hyper (hypo)-methlylated genes or differentially expressed genes often imply potential mechanistic relationships [4, 7–9].

In data mining field, the frequently co-occurring items are called frequent item sets. Their associated relationship (rules) can be efficiently mined via *Apriori* algorithm [10]. Originated from market basket data analysis, association rules mining (ARM) is a popular and well established method for discovering strong relationship between frequent items [11]. Alike to find frequent items and concurrent pattern in commercial datasets, we have proposed to identify frequent molecular alterations and combinations of these events from single or multiple omics data. Our OmicsARules package, which embedded with a new rule-interestingness measure *Lamda3*, can evaluate the association rules to identify biologically significant patterns.

## Implementation

**Fig. 1:**
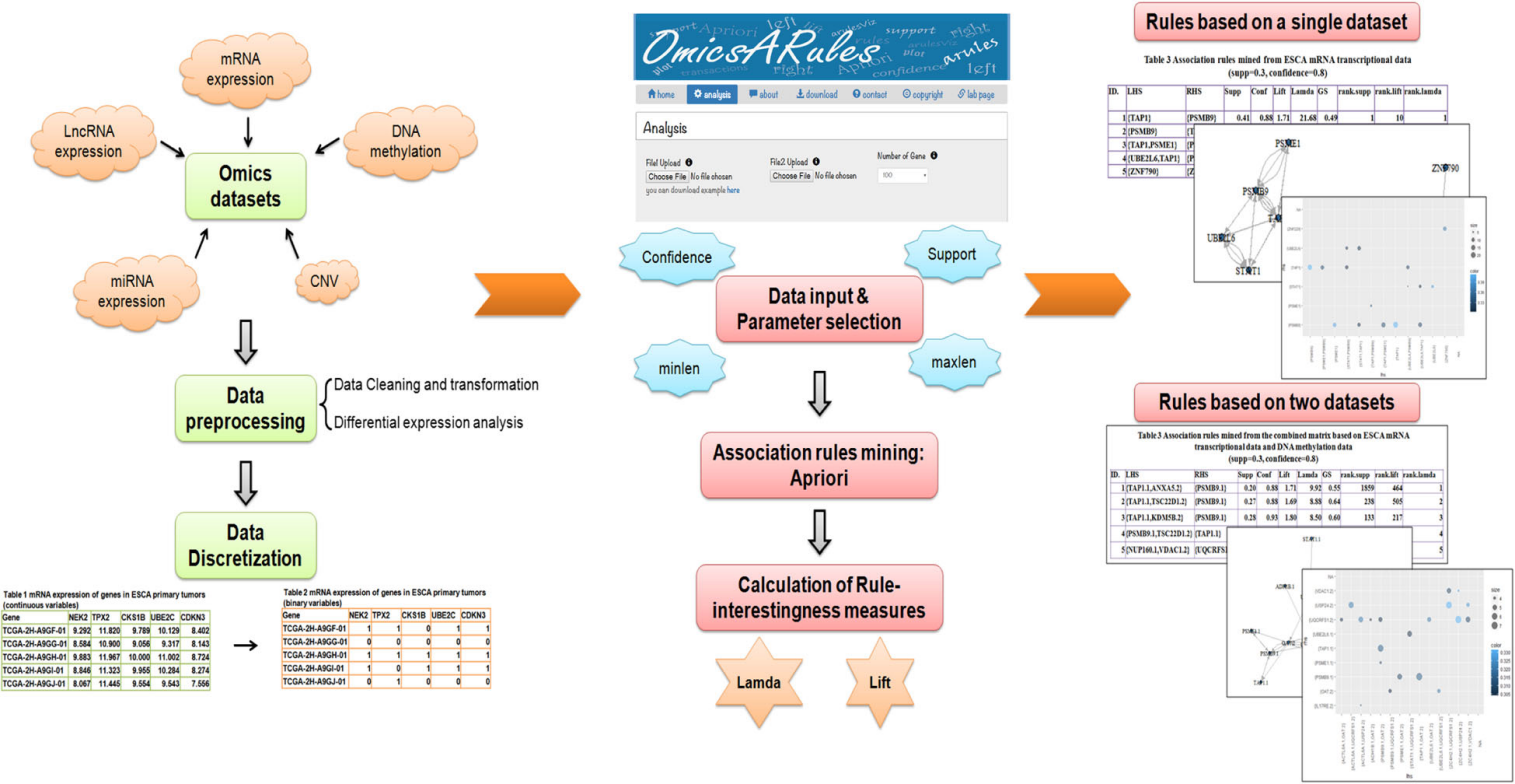
Schematic representation of the analysis pipeline performed by the OmicsARules

OmicsARules is implemented in R environment to analyze omics data sets under ARM framework (Fig. 1). Input data should be a matrix with continuous variables, such as mRNA profiling dataset or DNA methylation dataset. OmicsARules provides 5 simple processing methods to discretize the continuous values into binary matrix, which indicating the presence or absence of a molecular event in each sample. Users can mine and prioritize association rules, thresholding on several measures of significance and interestingness. The output from OmicsARules includes a table listed the found associated rules with significance and interestingness measures, and graphical presentations, which efficiently organize and visualize the identified rules for further exploration. Based on combination of various alteration spectrum simultaneously obtained from different sequencing platforms (i.e. both mRNA profiling dataset and DNA methylation dataset for the same group of patients), OmicsARules can identify the concordant changes among genes, which usually indicates broader biological implications.

### Association rules mining and its application in omics datasets

Let *I =* {*i*_1_*, i*_2_*,*. *.*.*, i*_n_} be a set of *n* binary attributes called items. Let *D =* {*t*_1_*, t*_2_*,*. *.*.*, t*_m_} be a set of transactions called the database. Each transaction in *D* has a unique transaction ID and contains a subset of the items purchased in *I*. A rule is defined as an implication of the form *X = > Y* where *X, Y* ⊆ *I* and *X ∩ Y =* ∅. The sets of items (for short, item-sets) *X* and *Y* are called antecedent (left-hand-side, LHS) and consequent (right-hand-side, RHS) of the rule, respectively. Frequent item-set, which is defined as the frequently co-occurring items, composes the association rule. Finding frequent item-set is a principal theme underlying identification of association rules. Utilizing a “bottom-up” approach, *Apriori* is a basic algorithm for identifying association rules [10]. It extends one item at a time to generate candidate frequent item-sets, and terminates if there is no further successful extensions to be identified. At each step, candidates having an infrequent sub-pattern are eliminated. Constraints on various measures of significance and interestingness of rules, such as *Support*, *Confidence* and *Lift*, could be used to rank the identified association rule (See Additional file 1 for formal definitions of these measures).

In a similar sense, items usually refer to genes in omics dataset. The frequent items could be significantly mutated genes, hypo−/hyper-methlylated genes and up−/down-expressed genes, etc., which occur more frequently than expected by random chance. Transaction indicates each independent patient sample. Notably, the frequent item-set, a set of co-occurrence between the interested genes, often implies potentially vital mechanistic connections [4, 7, 8]. To illustrate the concept, a small example from the gene expression profile was shown in Table 1. Rows correspond to each patient sample whereas columns correspond to the measured genes. Zero or one in the matrix indicates whether or not that gene is dys-regulated in that sample. In this case, the item-set is *I =* {NEK2,TPX2, CKS1B, UBE2C, CDKN3} and an example rule for this data could be {NEK2} *= >* {CDKN3}, which means, if the expression of NEK2 is altered, CDKN3 is also differentially expressed.

**Table 1:** An example of the application of ARM to omics datasets

Genes	NEK2	TPX2	CKS1B	UBE2C	CDKN3
Patient Samples					
TCGA-2H-A9GF-01	1	1	0	1	1
TCGA-2H-A9GG-01	0	0	0	0	0
TCGA-2H-A9GH-01	1	1	1	1	1
TCGA-2H-A9GI-01	1	0	1	1	1
TCGA-2H-A9GJ-01	0	1	0	0	0

### *Lamda3* is a novel measure indicating the interestingness of rules

ARM is supposed to be used on binary datasets, so continuous omics dataset should be transformed to the binary matrix before mining association rules. However, data transformation often results to information loss. Besides, the cutoff values used in transformation are arbitrary and could have a dominant affect on the performance of ARM. To overcome these obstacles, we proposed a novel rule-interestingness measure, *Lamda3* on basis of coordinated changes among genes. Suppose the input continuous matrix containing *M*_c_, which is of size *m* × *n*, where *m* denoted #sample and *n* for #gene. After data discretization, it will be transformed into a Boolean matrix, *M*_b_, containing ‘1’ indicating dys-regulation and ‘0’ representing insignificant change. Given an association rule, *Lamda3* is defined as the ratio of the association strength between LHS genes and RHS genes, to the average association strength between the LHS gene and other genes which are not included in that rule. For simplicity, we assumed here the identified association rule is *Z*, *A*= > *C*, i.e. only one gene *A* on the LHS, and one gene *C* on the RHS for each rule. Then firstly, according to *M*_b_, all the samples in *M*_c_ were divided into three parts, ${M}_{\mathrm{c}}^2$, ${M}_{\mathrm{c}}^1$ and ${M}_{\mathrm{c}}^0$. ${M}_{\mathrm{c}}^2$ contains samples with gene *A* to be “1” and simultaneously gene *C* to be “1” in the *M*_b_. ${M}_{\mathrm{c}}^1$ indicates samples having inconsistent changes of these two genes of this rule. Besides, ${M}_{\mathrm{c}}^0$ indicates samples with both *A* and *C* to be “0” in the *M*_b_. That is, ${M}_{\mathrm{c}}^2$ and ${M}_{\mathrm{c}}^0$ includes samples with consistent changes of these two genes in this rule, while the former matrix contains samples with both *A* and *C* are dys-regulated while the latter has normal genes. Secondly, the association strength between genes could be defined as sum of correlation significance in the ${M}_{\mathrm{c}}^2$ and ${M}_{\mathrm{c}}^0$. The corresponding *P* values of correlation measure can be calculated as follows:

$$ {P}_{A,C}^2\leftarrow cor\left(A,C\right)\ \mathrm{in}\ {M}_{\mathrm{c}}^2; $$

$$ {P}_{A,C}^0\leftarrow cor\left(A,C\right)\ \mathrm{in}\ {M}_{\mathrm{c}}^0.. $$

At the same time, the *P* values of correlations between gene *A* and the other genes in the matrix (except for *A* and *C*), were calculated in the ${M}_{\mathrm{c}}^2$ and ${M}_{\mathrm{c}}^0$ as follows:

$$ {P}^2\leftarrow \mathrm{median}\left({P}_{A,{g}_1}^2,{P}_{A,{g}_2}^2,\dots, {P}_{A,{g}_{\mathrm{i}}}^2,\dots \right),{g}_{\mathrm{i}}\in {M}_{\mathrm{c}}^2,\mathrm{but}\ne A,C; $$

$$ {P}^0\leftarrow \mathrm{median}\left({P}_{A,{g}_1}^0,{P}_{A,{g}_2}^0,\dots, {P}_{A,{g}_{\mathrm{i}}}^0,\dots \right),{g}_{\mathrm{i}}\in {M}_{\mathrm{c}}^0,\mathrm{but}\ne A,C; $$

where, ${P}_{A,{g}_{\mathrm{i}}}^2\leftarrow cor\left(A,{g}_{\mathrm{i}}\right)$ in the ${M}_{\mathrm{c}}^2$ and ${P}_{A,{g}_{\mathrm{i}}}^0\leftarrow cor\left(A,{g}_{\mathrm{i}}\right)$ in the ${M}_{\mathrm{c}}^0$. Then *Lamda3* was defined as,

$$ \mathrm{Lamda}3=\frac{\log_{10}\left({P}_{A,C}^2\right)+{\log}_{10}\left({P}_{A,C}^0\right)}{\log_{10}\left({P}^2\right)+{\log}_{10}\left({P}^0\right)}. $$

An example of calculating the proposed *Lamda3* was presented in Additional file 1.

## Results

### Lamda3 can select biologically relevant rules from single omic dataset

OmicsARules pipeline was in turn applied to single omic dataset such as mRNA RNA-seq or DNA methylation datasets from three types of cancers (BRCA, ESCA and LUAD). For each of the six datasets, the top 50 or 100 differentially changed genes were used for association rules mining. Then, we retrieved the top 20 rules ranked by various significance measures, and their average GS scores were estimated for evaluation of these rules [12].

**Fig. 2:**
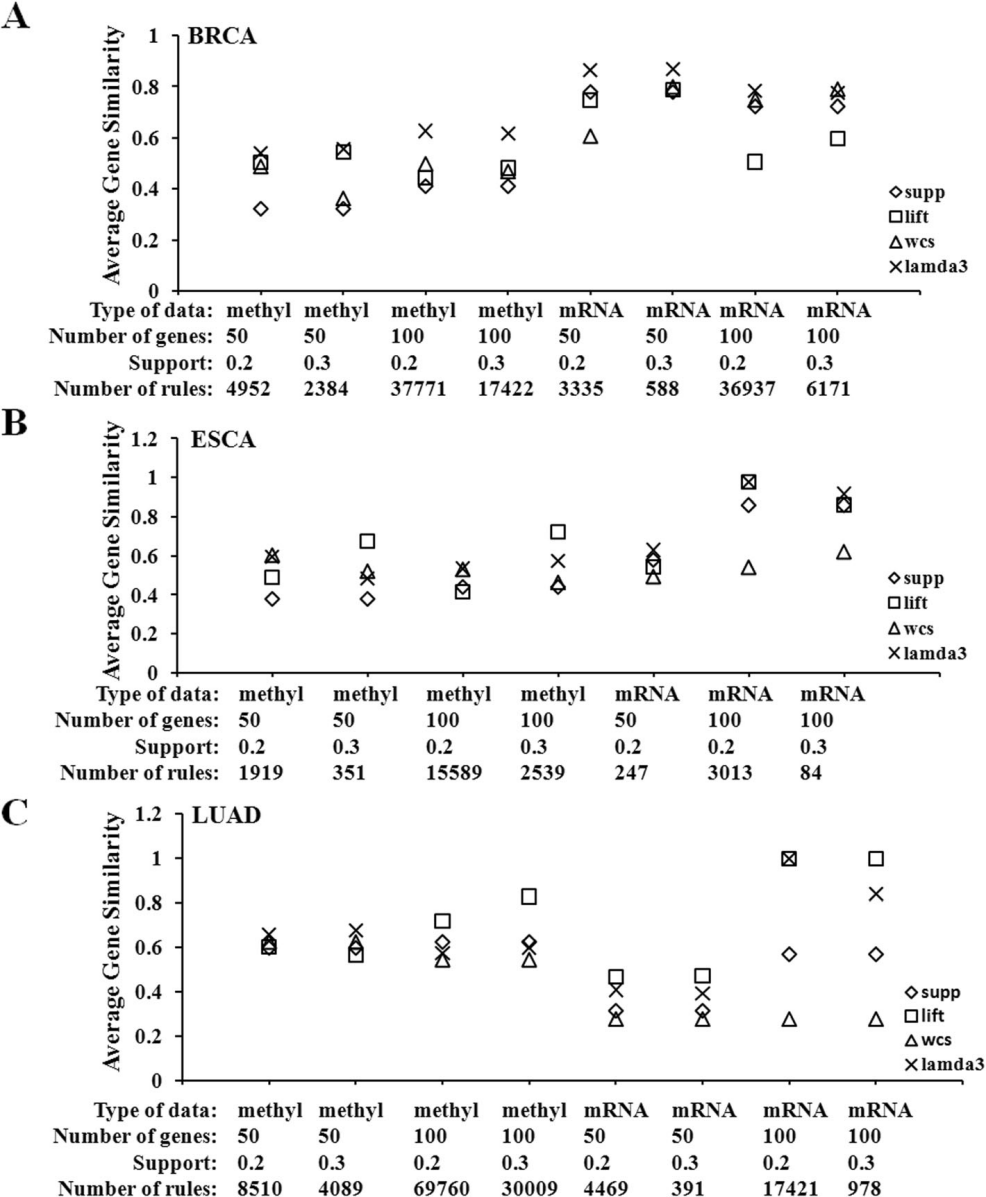
Quantitative assessment of the biological relationship for genes within the same association rule based on gene similarity score ARM was conducted for single omic dataset based on different parameter settings (**a**) BRCA, (**b**) ESCA and (**c**) LUAD. The top-ranked 20 rules identified by either *Support, Lift, wcs* or *Lamda3*, were extracted and the average gene similarity scores were calculated. Number of gene, *n* top-ranked abnormal gene used for ARM

As shown in Fig. 2a, in all comparisons from BRCA, *Lamda3* performs superiorly than the other three rule-interestingness measures. The top 20 rules (based on *n* = 50, Supp = 0.3, Conf = 0.8) ranked by either *Supp*, *Lift* or *Lamda3* from mRNA dataset or methylation dataset were shown in Additional file 2: Tables S2 and Table S3, respectively. It was noticed that these rules have similar values of *Conf*, *Supp* and *Lift*. But *Lamda3* could better differentiate them. Genes constituting the top 20-ranked rules by *Lamda3* in BRCA mRNA dataset, were all from HOXA cluster, namely HOXA3, HOXA4, HOXA5, HOXA7. HOX genes encode a highly conserved family of homeodomain-containing transcription factors that have crucial roles in specifying positional identity along the anterior–posterior body axis during embryogenesis [13]. According to their located chromosomes, the mammalian HOX genes have been classified into 4 clusters (HOXA, −B, −C, and -D). HOXA3, HOXA4, HOXA5 and HOXA7 are located on chromosome 7. Previous studies had indicated dys-regulated HOX gene expression in carcinogenesis and breast cancer metastasis. HOXA3 and HOXA7 were found to be down-regulated in the MDA-MB-231 cells compared to the non-malignant cells [14, 15]. Expression levels of HOXA3 and A5 were found to be significantly different between breast cancerous and normal tissues [16]. Loss of expression of p53 in human breast cancer was supposed to be primarily due to lack of expression of HOXA5 [17]. Although little is known about HOXA4 in breast cancer currently, the co-occurrence of HOXA3, A4, A5 and A7 in these rules identified an intimate expression relationship between HOXA4 and the other three HOXA genes, suggesting its potential role in carcinogenesis.

As seen in Fig. 2b, *Lamda3* also performs better than the other three rule-interestingness measures based on ESCA mRNA dataset. As for the ESCA methylation dataset, *Lamda3* had the better performing when *Support* is set to be 0.2, in contrast to *Support* = 0.3. Also observed in Fig. 2c of LUAD mRNA dataset, the performance of *Lamda3* and *Lift* are comparable, and both are better than the other two measures. Regarding the LUAD methylation dataset, *Lamda3* achieved the best performance when including only 50 genes, but was not as good as *Lift* when 100 genes were included. All the corresponding top-ranked 20 rules selected either by *Lamda3*, *Lift* or *Support* were shown in Additional file 2: Tables S4-S7.

Overall, the performance of *Lamda3* is superior to other measures for the mRNA datasets in all the three cancers. As for the methylation dataset, *Lamda3* and *lift* performed comparable and better than other measures. Therefore, *Lamda3* is capable to identify biologically significant rules.

### Lamda3 can identify biologically relevant rules from combined multi-omics datasets

**Fig. 3:**
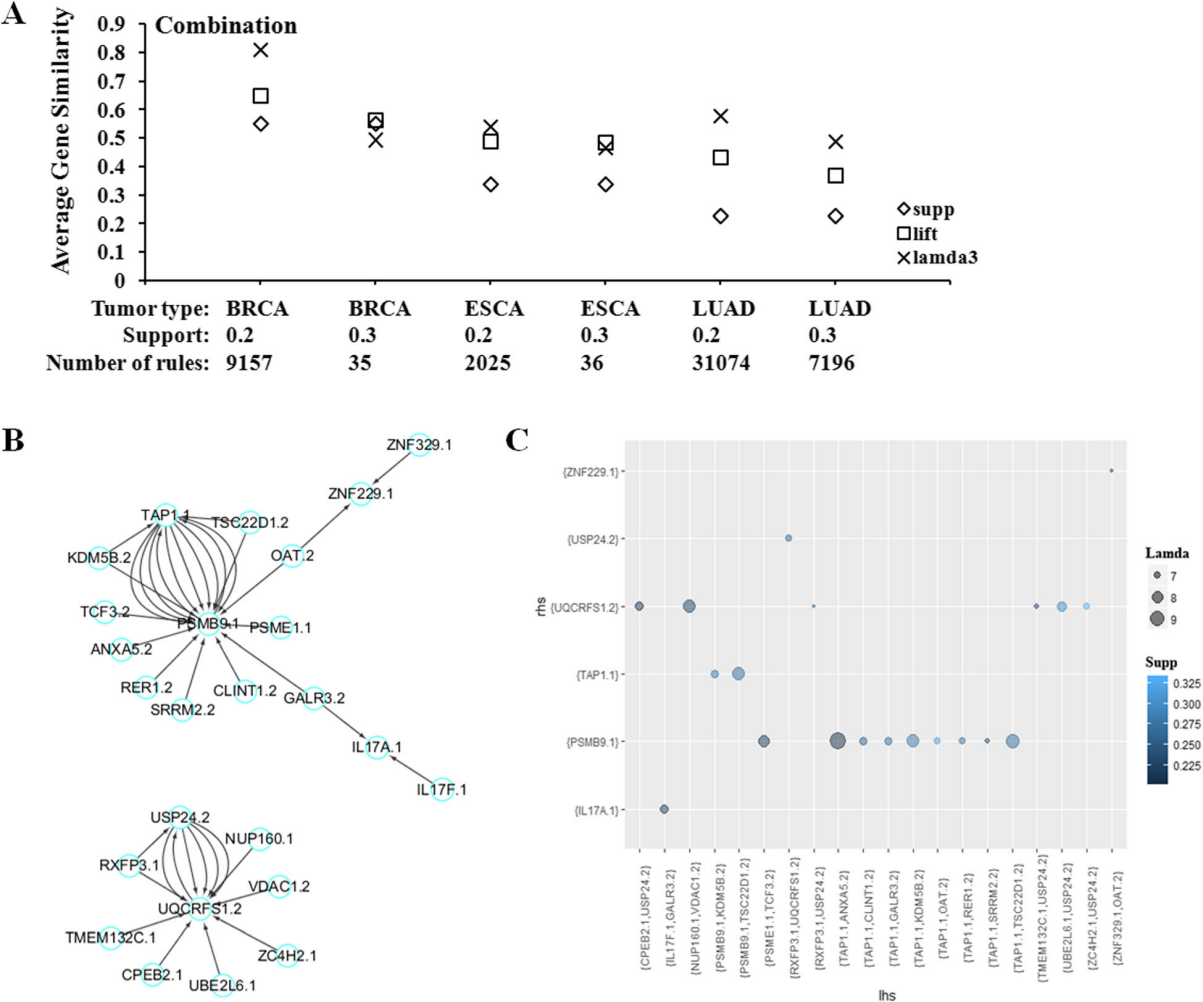
Quantitative assessment and graphical representation of association rules from multi-omics datasets. Based on different parameter settings, ARM was conducted for multi-omics datasets from BRCA, ESCA and LUAD. **a** The top-ranked 20 rules identified by either *Supp, Lift* and *Lamda3*, were extracted and the average gene similarity scores were calculated; **b** The representative network graph as well as (**c**) the group graph was shown for the top-ranked 20 rules by *Lamda3* from ESCA multi-omics datasets

Multi-omics experiments provide good opportunity to explore tumor formation and development via answering questions at systems level, such as how the genetic or epigenetic factors coordinate to drive the malignance in cancer? Here, for each cancer, DNA methylation and transcriptional data were collected from the same group of patients. After processing, the top-ranked 50 differentially expressed (DE) or differentially methylated (DM) genes were combined into one single matrix according to samples IDs and then subjected to ARM. The obtained rules were filtered to retain those which LHS and RHS contain genes from different omic datasets. Then these rules were ranked according to interestness measures, *Lift, Lamda3* as well as *Support*. Notably, *wcs* could not be estimated because of the lack of rank-based weights assigned to each gene under current circumstances. As indicated by GS scores in Fig. 3a, *Lamda3* is higher than other two measures in all these three cancer datasets based on Supp = 0.2. When support was set to be 0.3, the performance of *Lamda3* is still the best in LUAD datasets. Regarding the analysis of the BRCA and ESCA datasets based on Supp = 0.3, the average GS scores of rules ranked by these three measures were very close to each other. Overall, *Lamda3* can identify more biologically significant association rules from combined multi-omics datasets. The top-ranked 20 rules (based on Supp = 0.2, Conf = 0.8) mined from the three cancers were shown in Additional file 2: Tables S8-S10.

As shown in Fig. 3b, according to their association, genes in the top-ranked 20 rules from ESCA constituted two subsets of network. PSMB9 mRNA change is the hub of one sub-network. PSMB9 locates in the class II region of the MHC (major histocompatibility complex) and encodes the proteasome subunit. In previous studies, there is no evidence of positive relationship between PSMB9 and ESCA, but PSMB9 has been suggested to be potential targets for the diagnosis and therapy for several other cancers, such as cutaneous squamous cell carcinoma and cervical cancer [18, 19]. As shown in Fig. 3b and c, the association rules {TAP1.1,ANXA5.2}= > {PSMB9.1} and {TAP1.1,KDM5B.2}= > {PSMB9.1} were identified by *Lamda3*. These two rules suggested a combined mechanism between TAP1 expression and DNA methylation of ANXA5/KDM5B, which leads to the abnormal expression of PSMB9 in ESCA. ANXA5 is a phospholipase A2 and protein kinase C inhibitory protein with calcium channel activity and it plays a role in cellular signal transduction, inflammation, growth and differentiation [20]. An association between ANXA5 and several cancers has been assumed [20]. KDM5B is the specific demethylase of histone H3 lysine 4 (H3K4), and its’ up-regulation can reduce H3K4 methylation level. In previous studies, a crucial role of histone lysine methylation in the epigenetic regulation of eukaryotic genes has been demonstrated, which suggested histone methylation disorders can cause cancer [21, 22]. KDM5B also involves in ESCA development and progression [23]. Here, a potential mechanistic association between the DNA methylation of KDM5B and the transcript levels of PSMB9 was proposed by *Lamda3*, which provided a research clue to be tested further. Interestingly, one recent paper found that, DNMT inhibitors (that removed DNA methylation) up-regulate expression of the antigen processing and presentation molecules, including PSMB9 at the RNA and protein level in a wider range of colon and ovarian cancer cell lines [24].

These two rules also showed an association of PSMB9 and TAP1 on the transcript levels. TAP1, a member of the superfamily of ATP-binding cassette (ABC) transporters, is involved in the pumping of degraded cytosolic peptides into the membrane-bound compartment. Loss of TAP1 has been reported to render some tumor cells to escape the immune surveillance and contribute to the clinical course of esophageal cancer [25]. Again, the identified rules pinpoint a mechanistic link between TAP1 and PSMB9, thus provide testable hypothesis.

## Discussion

Multiple molecular events are responsible for the initiation and progression of diseases. Therefore, it is a key issue to identify the recurrent aberrations and associated changes at multi-modal data level. A standard approach is exploratory analysis of the interested cancers by querying a gene list against all available omics data [26, 27]. Also others jointly modeled individual alterations that arise from single platform over biological networks and pathway [28, 29]. Although these methods provided novel insights, few of them explicitly found and assessed the recurrent co-occurring aberrations across omics datasets. Besides, these tools usually involved sophisticated statistical modeling and scripts programming, thus have no easy-to-use access to biologists.

In this study, we applied the association mining for omics datasets. Especially, the newly developed interestness measure *Lamda3* minimized the loss of information due to dichotomization, achieved better biological significance over other rule-ranking measures. Besides, OmicsARules searches for the concurrent pattern among frequent aberrations from multiple omics datasets, thus to better illustrate the underlying common mechanism.

## Conclusions

OmicsARules package will be regularly updated and optimized to handle larger cancer datasets. We concluded that OmicsARules enables a new dimension to interpret the observed aberrations and regulation mechanism across high throughput platforms.

## Methods

### Data source and preprocessing

TCGA omics datasets of breast invasive carcinoma (BRCA), esophageal carcinoma (ESCA) and lung adenocarcinoma (LUAD) were downloaded. Each dataset includes both RNA sequencing data and DNA methylation data from the same cohort of patients. General information of these datasets was summarized in Additional file 2: Table S1.

Above datasets were subjected to OmicsARules to find biologically significant association rules. Before that, the raw data went through several preprocess steps, including removal of genes with missing values, differential expression analysis and discretization. More details are presented in Additional file 1.

### Application of OmicsARules on the cancer omics datasets

Single omic dataset can input directly to find association rules after data preprocessing. Regarding multi-omics datasets, each dataset was separately subjected to the preprocess step such as differential expression analysis and discretization. Then these two binary datasets were combined according the sample IDs, and then subjected to association rule mining by OmicsARules. In order to discriminate sources of genes, suffix ‘.1’ or ‘.2’ was added behind the gene symbol, thus the former indicated genes were present in the mRNA dataset; and the latter indicated genes were from the methylation data. Finally, rule-interestingness measures, namely *Lift* and *Lamda3*, as well as *Wcs* (weighted condensed support) [30], were calculated to rank the rules.

### Quantitative assessment of the biological significance of the identified rules

In order to assess and compare the biological significance of the rules identified by above interestingness measures, we calculated the annotated functional similarity of Gene Ontology (GO) terms, to evaluate the biological connection between gene(s) on the LHS and these on the RHS. Gene functional similarities (GS) between genes were computed with R-package ‘GOsim’ [12].

### Availability and requirements

Project name: OmicsARules.

Project home page: https://github.com/BioinformaticsSTU/OmicsARules

Operating system(s): Linux, Microsoft Windows.

Programming language: R.

Other requirements: *R* > =3.3.2.

License: MIT License.

Any restrictions to use by non-academics: No restrictions.

## Supplementary information

**Additional file 1.** Description of R package development, datasets preprocessing and assessment of the biological significance of the identified rules.

**Additional file 2: Table S1.** General information of three real datasets downloaded from TCGA. **Table S2.** Top 20 rules identified from BRCA mRNA dataset. **Table S3.** Top 20 rules identified from BRCA DNA methylation. **Table S4.** Top 20 rules identified from ESCA mRNA dataset. **Table S5.** Top 20 rules identified from ESCA DNA methylation dataset. **Table S6.** Top 20 rules identified from LUAD mRNA dataset. **Table S7.** Top 20 rules identified from LUAD DNA methylation dataset. **Table S8.** Top 20 rules identified from the combined BRCA mRNA and DNA methylation datasets. **Table S9.** Top 20 rules identified from the combined ESCA mRNA and DNA methylation datasets. **Table S10.** Top 20 rules identified from the combined LUAD mRNA and DNA methylation datasets.

## Data Availability

The OmicsARules package together with its installation guide, are freely accessible at https://github.com/BioinformaticsSTU/OmicsARules. Level 3 DNA methylation profiles as well as corresponding level 3 RNA sequencing data for breast invasive carcinoma, esophageal carcinoma and lung adenocarcinoma, were selected and downloaded from Genomic Data Common Data Portal (http://portal.gdc.cancer.gov/). For the convenience of users, we have uploaded the datasets on OneDrive (https://stumail-my.sharepoint.cn/:f:/g/personal/d_z_chen_stu_edu_cn/ErLfzFLiee9PjUEox0iyULEBIsE4SEwu7BVa8cYI8nQpYA?e=wic3GT).

## Abbreviations

ARM: Association Rules Mining
BRCA: BReast invasive CArcinoma;
DE: Differentially Expressed gene
DM: Differentially Methylated gene
ESCA: ESophageal CArcinoma
GO: Gene Ontology
LHS: Left-hand-side
LUAD: LUng ADenocarcinoma
MHC: Major Histocompatibility Complex
RHS: Right-hand-side
