# Supplementary material for: OmicsARules: a R package for integration of multi-omics datasets via association rules mining
Source: BMC Bioinformatics. 2019 Nov 8;20:554. doi: 10.1186/s12859-019-3171-0 (PMC6839229; doi:10.1186/s12859-019-3171-0)
Supplement: Supplementary file 1 — Additional file 1. Description of R package development, datasets preprocessing and assessment of the biological significance of the identified rules. [file 12859_2019_3171_MOESM1_ESM.docx]

Supplementary Information

Part One: OmicsARules package development

**Frequent itemsets and association rule**

Mining association rules is originally utilized for market basket data analysis to discover interesting relations between variables in large databases [[1](#_ENREF_1)].

Let ***I =* {*i _1_ , i* _2_ *, . . . , i* _n_ }**be a set of n binary attributes called *items*. Let ***D =* {*t* _1_ *, t* _2_ *, . . . , t* _m_ }** be a set of transactions called the *database*. Each transaction in D has a unique transaction ID and contains a subset of the items in I. A *rule* is defined as an implication of the form ***X => Y*** where ***X, Y ⊆ I and X ∩ Y = ∅***. The sets of items (for short itemsets) X and Y are called *antecedent* (left-hand-side or LHS) and *consequent* (right-hand-side or RHS) of the rule. Finding frequent itemsets is a principal theme underlying identification of association rules. In data mining field, frequent itemsets are defined as the frequently co-occurring items, and their associated relationship composes the association rule.

To illustrate the concepts, a small example from the supermarket domain was used as an example. The set of items is ***I = {milk, bread, butter, beer}*** and a small database containing the items is shown in Table 1. An example rule for the supermarket could be ***{milk, bread} -> {butter}*** meaning that if milk and bread are bought, customers also buy butter.

**Table 1 An example supermarket database with five transactions.**

| transaction ID | items |
| --- | --- |
| 1 | milk, bread |
| 2 | bread, butter |
| 3 | beer |
| 4 | milk, bread, butter |
| 5 | bread, butter |

Here, in the case of omics data, items usually refer to genes. Frequent items refer to genes measured in mutations, DNA methylation or mRNA expression, which occur more frequently than expected by random chance. Transaction indicates each independent sample. The frequent items could be significantly mutated genes, hypo- or hyper- methlylated genes and differentially expressed genes, etc. Notably, frequent itemset, that is a set of co-occurrence between these interested genes, often implies potentially vital mechanistic connections [[2-4](#_ENREF_2)]. In order to identify biologically significant rules, OmicsARules developed a new rule-interestingness measure *Lamda3* to prioritize the association rules obtained from classical Apriori algorithm mining [[5](#_ENREF_5)].

**Use of OmicsARules**

Before utilizing OmicsARules, the input file should fulfill the following format: data being separated by tab key, the column repressing each gene, and the row indicating each sample. The row names should be unique patient ID. The column names should be unique gene symbols, gene IDs etc. For each dataset, the matrix including continuous variables and the corresponding boolean matrix after dichotomization should be simultaneously used. (An example of uploading txt files were shown in Table 2 and Table 3).

**Table 2 An example of uploading the txt file containing continuous variables.**

| **Patient Samples** | **NEK2** | **TPX2** | **CKS1B** | **UBE2C** | **CDKN3** |
| --- | --- | --- | --- | --- | --- |
| **TCGA-2H-A9GF-01** | **6.48** | **6.00** | **12.54** | **5.74** | **4.38** |
| **TCGA-2H-A9GG-01** | **6.67** | **5.87** | **6.04** | **5.10** | **4.81** |
| **TCGA-2H-A9GH-01** | **6.94** | **7.43** | **7.35** | **6.09** | **5.19** |
| **TCGA-2H-A9GI-01** | **8.97** | **8.14** | **8.92** | **7.71** | **6.53** |
| **TCGA-2H-A9GJ-01** | **8.92** | **8.00** | **9.64** | **7.45** | **6.50** |

**Table 3 An example of uploading the txt file containing binary variables.**

| **Patient Samples** | **NEK2** | **TPX2** | **CKS1B** | **UBE2C** | **CDKN3** |
| --- | --- | --- | --- | --- | --- |
| **TCGA-2H-A9GF-01** | **1** | **1** | **0** | **1** | **1** |
| **TCGA-2H-A9GG-01** | **1** | **1** | **1** | **1** | **1** |
| **TCGA-2H-A9GH-01** | **1** | **1** | **1** | **1** | **1** |
| **TCGA-2H-A9GI-01** | **0** | **0** | **0** | **0** | **0** |
| **TCGA-2H-A9GJ-01** | **0** | **0** | **0** | **0** | **0** |

Users should first identify the interesting genes according to their own measures. For example, for mRNA profiling data, the genes can be selected and sorted by ***P*** values from differential expression analysis. OmicsARules provides five cutoff values to discretize the continuous values into binary matrix, namely mean, median, P25 (the upper quartile), P75 (the lower quartile), and del-outliers (mean after deleting outliers). According to the user's choice, OmicsARules calculates one of these cutoff values in each column, and if the values in each gene of a particular sample larger than the cutoff value, this value would be transformed into "1", otherwise, "0" is used.
 OmicsARules is supposed to use the top 500 genes from the uploading dataset for association rules analysis. Therefore, if the dataset contains more than 500 genes, you are required to sort these genes according to a certain score, which measure the importance of genes. This score could be *P* value, *t* statistic or AUC value after differentially expression analysis or diagnostic test.
 Constraints on various measures of significance and interest of rules could be used to rank each identified association rule. The following measures have been implemented in R package arules and arulesViz [[1](#_ENREF_1), [6](#_ENREF_6)].

**Support** of an itemset X is defined as the proportion of transactions in the dataset which contain the itemset. For example, in Table 1, the itemset {milk, bread} has a support of 2/5=0.4 since it occurs in 40% of all transactions (2 out of 5 transactions). Here, in the omics data, this 'support' measure indicates the proportion of patients in the dataset which contained the 'changed' pattern of a set of genes.

**Confidence** of a rule is defined as conf(X => Y ) = supp(X ∪ Y )/supp(X). Confidence can be interpreted as an estimate of the probability P (Y |X) of finding the RHS of the rule in transactions under the condition that these transactions also contain the LHS. For example, the rule {milk, bread} => [[7](#_ENREF_7)] has a confidence of 0.2/0.4 = 0.5 in the database in Table 1, which means that for 50% of the transactions containing milk and bread the rule is correct. In the omics data, the 'confidence' measure indicates that, in all the patients with the 'changed' pattern of gene set X, the proportion of patients accompany the 'changed' gene Y in the form of rule {X => Y}.
 **Lift** is another practical solution to narrow down the number of rules, in the situation of too many association rules found satisfying the support and confidence constraints. The lift of a rule is defined as lift(X => Y ) = supp(X ∪ Y )/(supp(X)supp(Y )), and can be interpreted as the deviation of the support of the whole rule from the support expected under independence given the supports of the LHS and the RHS. For example, in Table 1, the rule {milk, bread} -> [[7](#_ENREF_7)] has a lift of 0.2/(0.2×0.6) = 1.67 in the database in Table 1, which means that the realistic occurrence rate of this rule is 1.67 times more than the co-occurrence rate calculated under independence. In the omics data, lift measure indicated the possibility of co-occurrence between gene or gene set X and gene Y in the form of rule{X => Y}.

**Lamda3** Association rule mining is supposed to be used on binary datasets, so continuous omics dataset should be transformed to the binary matrix before mining association rules. However, data transformation often results to information loss. Besides, the cutoff values used in transformation are arbitrary and could have a dominant affect on the performance of ARM. Therefore, we proposed a novel rule-interestingness measure, Lamda3 on basis of coordinated changes among genes. Suppose the input continuous matrix containing Mc, which is of size m×n, where m denoted #sample and n signified #gene. After data discretization, it will be transformed into a boolean matrix, Mb. Given an association rule, Lamda3 is defined as the ratio of the association strength between genes on the LHS and the RHS, to the average association strength between the gene(s) on the LHS and others in the data matrix but not in that rule. Given an association rule Z has three genes, A, B=> C, then correlation analyses between A (or B) and C were performed as follows,

$P_{A,C}^{2}\leftarrow cor\left( A,C \right)$ in $M_{c}^{2}$;

$P_{B,C}^{2}\leftarrow cor\left( B,C \right)$ in $M_{c}^{2}$

$P_{A,C}^{0}\leftarrow cor\left( A,C \right)$ in $M_{c}^{0}$;

$P_{B,C}^{0}\leftarrow cor\left( B,C \right)$ in $M_{c}^{0}$;

Meanwhile, the correlation analyses between gene A (or B) and the other genes in the matrix (except for A, B and C), were performed to obtain the P values in the $M_{c}^{2}$ and $M_{c}^{0}$, respectively, shown as follows:

$P_{A}^{2}\leftarrow median\left( P_{A,g_{1}}^{2}, P_{A,g_{2}}^{2},\ldots,P_{A,g_{i}}^{2},\ldots\right)$, $g_{i}\in M_{c}^{2}$, but $\neq A, B and C$;

$P_{A}^{0}\leftarrow median\left( P_{A,g_{1}}^{0}, P_{A,g_{2}}^{0},\ldots,P_{A,g_{i}}^{0},\ldots\right)$, $g_{i}\in M_{c}^{2}$, but $\neq A, B and C$;

$P_{B}^{2}\leftarrow median\left( P_{B,g_{1}}^{2}, P_{B,g_{2}}^{2},\ldots,P_{B,g_{i}}^{2},\ldots\right)$, $g_{i}\in M_{c}^{2}$, but $\neq A, B and C$;

$P_{B}^{0}\leftarrow median\left( P_{B,g_{1}}^{0}, P_{B,g_{2}}^{0},\ldots,P_{B,g_{i}}^{0},\ldots\right)$, $g_{i}\in M_{c}^{2}$, but $\neq A, B and C$;

Consequently, Lamda3 for Z: A, B=>C was calculated as follows.

$Lamda3=\frac{\log_{10} \left( P_{A,C}^{2} \right)+\log_{10} \left( P_{B,C}^{2} \right)+\log_{10} \left( P_{A,C}^{0} \right)+\log_{10} \left( P_{B,C}^{0} \right)}{\log_{10} \left( P_{A}^{2} \right)+\log_{10} \left( P_{A}^{0} \right)+\log_{10} \left( P_{B}^{2} \right)+\log_{10} \left( P_{B}^{0} \right)}$.

It should be mentioned that, if the sample size of $M_{c}^{2}$ and/or $M_{c}^{0}$ is no more than 2, then the corresponding **P** values for the correlation analyses were set to be 1. An example of calculating the proposed Lamda3 was presented in Supplementary Figure 1.


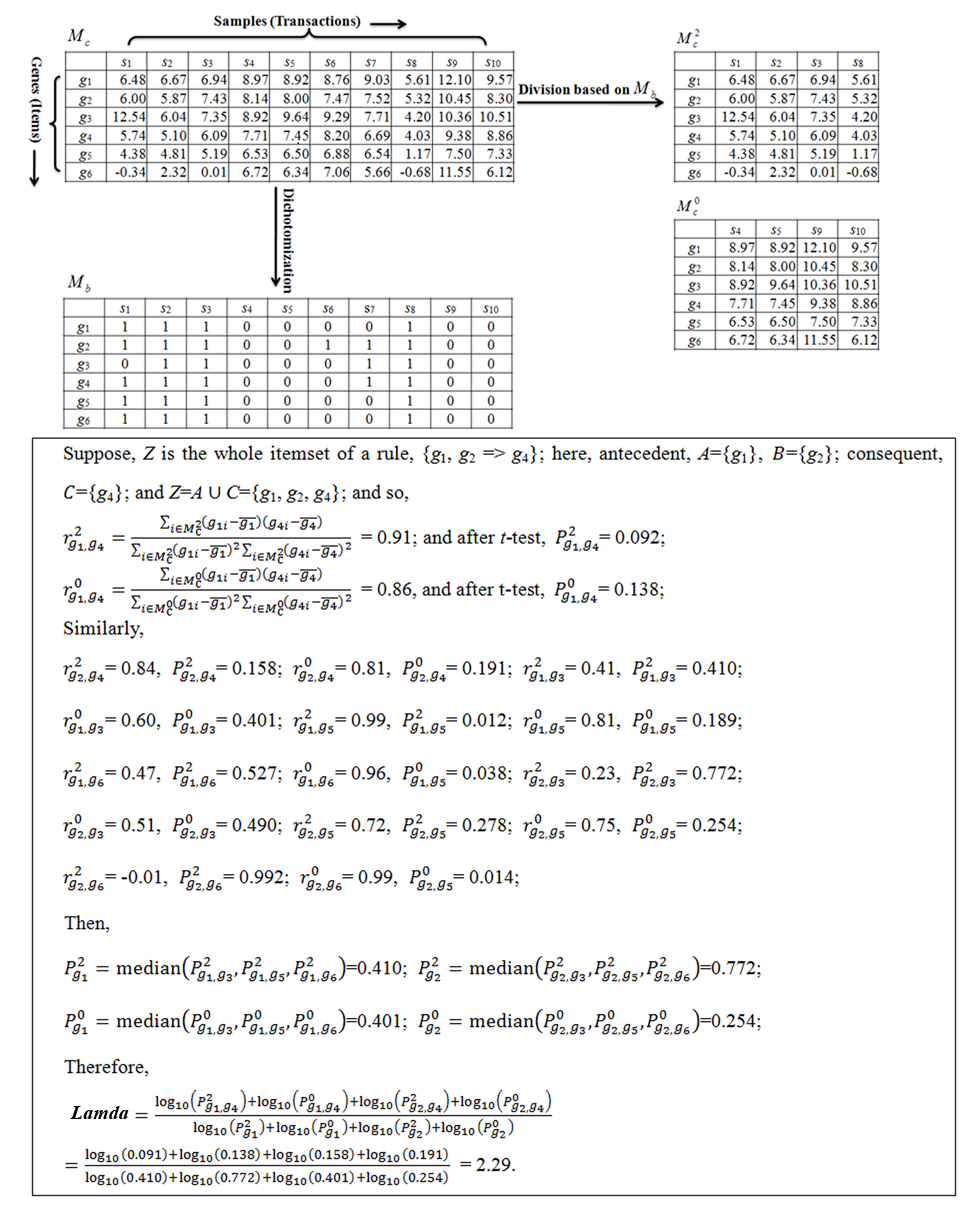


Part Two: BRCA, ESCA and LUAD datasets preprocessing

**Data source**

Level 3 DNA methylation profiles determined using an HumanMethylation450 array, as well as corresponding level 3 RSEM-normalized RNA sequencing data determined by IlluminaHiSeq_RNASeqV2 arrays (measured using RSEM software; version 1.2.31) [[8](#_ENREF_8)] were downloaded from TCGA (accessed: January 2016; known as the Genomic Data Common Data Portal; [http://portal.gdc.cancer.gov/](https://portal.gdc.cancer.gov/)). Three types of cancer, namely breast invasive carcinoma (BRCA), esophageal carcinoma (ESCA) and lung adenocarcinoma (LUAD), were selected. General information of these datasets was summarized in **Supplementary Table 1 of Additional Files 2**.

**Data preprocessing**

In this article, the real datasets downloaded from TCGA were subject to OmicsARules to find biologically significant association rules. Before that, the raw data went through several preprocess steps, including removal of genes with missing values, differential expression analysis and discretization.

**A. Data cleaning and transformation**

Microarray technique is a useful tool for measuring expression levels of tens of thousands of genes at the same time across different experimental and control samples. At first, genes or probes with missing values in any of the samples were pre-filtered. Secondly, as for mRNA data, the normalized values were base-2 logarithm transformed. Regarding DNA methylation data, β values calculated the percentage of DNA methylation in the tissue samples at each CpG probe, and ranged from 0 (unmethylated) to 1.0 (fully methylated); here, the M-value [logit(β)] was used instead of the β-value to calculate test-statistics. The association between M- and β-values were determined as follows, M-value= Log2[β-value/(1-β-value)].

**B. Differential expression analysis**

Each dataset included both tumors and adjacent normal tissues. In order to identify differentially expressed genes (DE) or differentially methylated genes (DM), student's t test was used with a function 't.test' from the R-package 't.test'. Benjamini & Hochberg (BH) was used to adjust P values due to the multiple testing [Controlling the false discovery rate: a practical and powerful approach to multiple testing, 1995]. In fact, probably because of higher variance of genes/probes inside the group than that between the groups, sometimes significant P-value turned out to be actually insignificant. Thus, it is needed to further examine the capacity of genes/probes to distinguish tumors from adjacent normal tissues. Here, we used a function 'roc' in R-package 'pROC' to create the ROC curve for each gene/probe, and then calculate the AUC (area under curve). Consequently, genes with adjusted P values (from t-test) <0.01, as well as with AUC≥0.8 were identified as DE/DM, and then ranked w.r.t. their P values.

**C. Data discretization**

Suppose Mc [m, n] is the input omics data matrix with continuous variables. Here, m denotes samples, and n denotes genes. Discretization of the input data matrix is mandatory for applying ARM. For the discretization purpose, we set the mean expression level of one specific gene to be the cutoff value. Before that, outliers were detected and removed using the function 'grubbs.test' in R-package 'outliers'. If there was no more outliers, the mean was then calculated, which is used to divide all the samples. According to the differential expression analysis, compared to that in normal tissues, if one gene was up-regulated/hyper-methylated in tumors, then tumors with expression levels larger than the mean was set to be 1. Similarly, if one gene was down-regulated/hypo-methylated, then tumors with expression values lower than the mean was set to be 1. In either case, expression levels of other tumor samples were set to be 0. Consequently, Mb was the resulting boolean matrix. According to Mb, “1” denoted dys-regulated gene expression or methylation, and “0” denoted insignificant change. Similar to that in ARM, “1” and “0” signified presence and absence of some item (gene) in some transaction (sample). An example of the discretization and post-discretization processes was shown in **Supplementary Figure 2**.


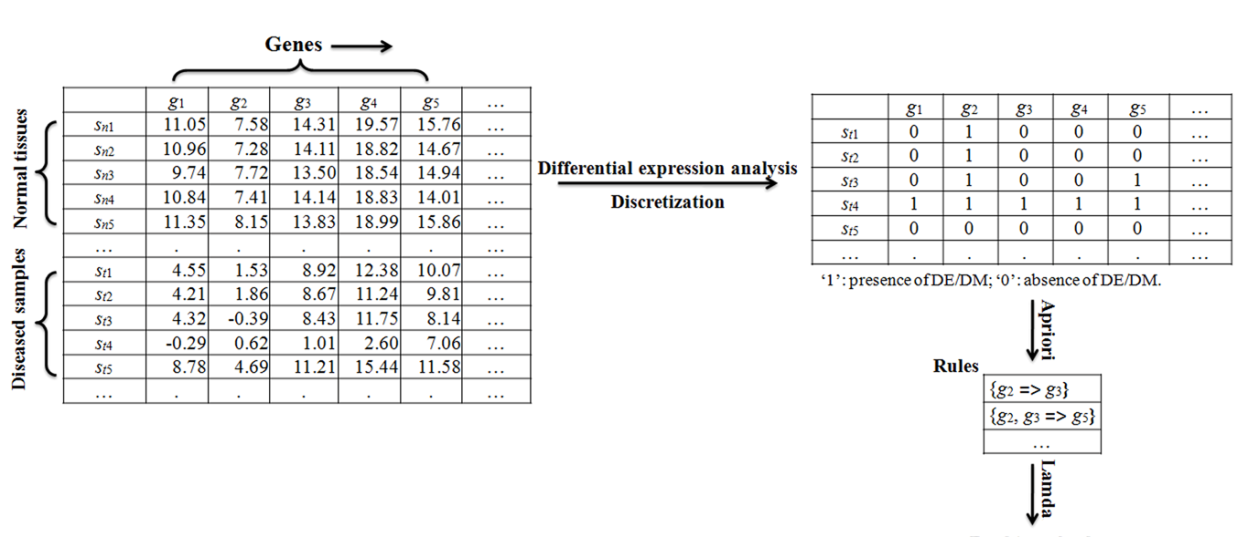


Part Three: Quantitative assessment of the biological

significance of the identified rules

Gene functional similarity on the basis of the similarity of Gene Ontology (GO) terms, was used to evaluate the biological significance between genes. Here, the maximum pair-wise GO term similarity was calculated[[9](#_ENREF_9)]. Given two genes A and B annotated with GO terms c*i* (i=1, ..., N) and c*j* (j=1, ..., M), the functional similarity between A and B was defined as:

$funSimMax\left( A,B \right)=max\left\{ \frac{1}{M}\sum_{j=1}^{M} \max_{1\leq i\leq N} s_{ij}, \frac{1}{N}\sum_{i=1}^{N} \max_{1\leq j\leq M} s_{ij} \right\}$ , where

$s_{ij}=\mathrm{sim}_{\mathrm{Rel}}\left( {GO}_{i}^{A}, {GO}_{j}^{B} \right)$, $\forall i\in\left\{ 1,\ldots,N \right\}$; $\forall j\in\left\{ 1,\ldots,M \right\}$.

The funSim score lies in the interval [0, 1]. The similarity matrix S contains all pair-wise similarity values of mappings of gene A and mappings of gene B. Here, we used the ‘Relevance similarity’ algorithm [[9](#_ENREF_9)]. If c1 is one of GO terms mapped by A, and c2 is another GO term mapped by B, then the similarity of these two terms was defined as follows:

$s_{ij}=\mathrm{sim}_{\mathrm{Rel}}\left( c_{1}, c_{2} \right)=\max_{c\in S(c_{1},c_{2})} \left( \frac{2\cdot\log p\left( c \right)}{\log p\left( c_{1} \right)+\log p\left( c_{2} \right)}\cdot\left( 1-p\left( c \right) \right) \right)$, where

$p\left( c \right)=freq\left( c \right)/ freq\left( root \right)$

$\mathrm{freq}\left( c \right)=anno\left( c \right)+\sum_{h\in children\left( c \right)} \mathrm{freq}\left( h \right)$

$S\left( c_{1},c_{2} \right)$ was the set of common ancestors of terms c_1_ and c_2_. $\mathrm{freq}\left( c \right)$ denoted the frequency of a GO term and $\mathrm{freq}\left( root \right)$was the frequency of the root term. $\mathrm{anno}\left( c \right)$ was the number of genes annotated with this term in the database. $\mathrm{children}\left( c \right)$ was the set of child nodes of term c. By definition, it could be concluded that, the larger average gene similarity was, the more biologically significant rules.

**Reference**

1. Hahsler M, Grun B, Hornik K: **arules, A Computational Environment for Mining Association Rules and Frequent Item Sets**. *Journal of Statistical Software* 2005, **14**(15):1-25.

2. Bailey P, Chang DK, Nones K, Johns AL, Patch AM, Gingras MC, Miller DK, Christ AN, Bruxner TJ, Quinn MC *et al*: **Genomic analyses identify molecular subtypes of pancreatic cancer**. *Nature* 2016, **531**(7592):47-52.

3. George J, Lim JS, Jang SJ, Cun Y, Ozretic L, Kong G, Leenders F, Lu X, Fernandez-Cuesta L, Bosco G *et al*: **Comprehensive genomic profiles of small cell lung cancer**. *Nature* 2015, **524**(7563):47-53.

4. Villanueva J, Infante JR, Krepler C, Reyes-Uribe P, Samanta M, Chen HY, Li B, Swoboda RK, Wilson M, Vultur A *et al*: **Concurrent MEK2 mutation and BRAF amplification confer resistance to BRAF and MEK inhibitors in melanoma**. *Cell Rep* 2013, **4**(6):1090-1099.

5. Agrawal R, Imielinski T, A. S: **Mining association rules between sets of items in large databases**. In: *Proceedings of the ACM SIGMOD International Conference on Management of Data: 1993; Washington DC*; 1993: 207-216.

6. Hahsler M, Chelluboina S: **Visualizing Association Rules: Introduction to the R-extension Package arulesViz**. *R package* 2016.

7. Cancer Genome Atlas Research N, Ley TJ, Miller C, Ding L, Raphael BJ, Mungall AJ, Robertson A, Hoadley K, Triche TJ, Jr., Laird PW *et al*: **Genomic and epigenomic landscapes of adult de novo acute myeloid leukemia**. *N Engl J Med* 2013, **368**(22):2059-2074.

8. Li B, Dewey CN: **RSEM: accurate transcript quantification from RNA-Seq data with or without a reference genome**. *BMC Bioinformatics* 2011, **12**:323.

9. Schlicker A, Domingues FS, Rahnenfuhrer J, Lengauer T: **A new measure for functional similarity of gene products based on Gene Ontology**. *BMC Bioinformatics* 2006, **7**:302.
